# Scar Ectopic Pregnancy as an Uncommon Site of Ectopic Pregnancy: A Case Report and Literature Review

**DOI:** 10.3390/life13112151

**Published:** 2023-10-31

**Authors:** Mamoun Elawad, Suad Zaki Hamed Alyousef, Njoud Khaled Alkhaldi, Fayza Ahmed Alamri, Hanadi Bakhsh

**Affiliations:** 1 Obstetrics & Gynecology Department, King Abdullah Bin Abdulaziz University Hospital, Princess Nourah bint Abdulrahman University, Riyadh 11671, Saudi Arabia; mmelawad@kaauh.edu.sa; 2 General Medicine and Surgery, College of Medicine, Hail University, Hail 2440, Saudi Arabia; suad.zaki4@gmail.com; 3 Clinical Sciences Department, College of Medicine, Princess Nourah bint Abdulrahman University, Riyadh 11671, Saudi Arabia; nks.alkhaldi@gmail.com (N.K.A.); iifoii.34@gmail.com (F.A.A.); 4 Department of Obstetrics and Gynecology, King Abdullah Bin Abdulaziz University Hospital, Princess Nourah bint Abdulrahman University, Riyadh 11671, Saudi Arabia

**Keywords:** cesarean scar pregnancy, ectopic pregnancy, diagnosis, management, surgical management, hysteroscopy, laparoscopy, laparotomy, fertility

## Abstract

A cesarean scar pregnancy is a rare type of ectopic pregnancy that occurs when a fertilized egg implants in the scar from a previous cesarean section. It is a serious condition that can lead to significant morbidity and mortality if not managed promptly and appropriately. In this literature review and case report, we discuss the etiology, diagnosis, and management of cesarean scar pregnancy. We conducted a comprehensive search of relevant literature using electronic databases and included studies that reported on the diagnosis and management of cesarean scar pregnancy. We also present a case report of a patient with cesarean scar pregnancy who was managed surgically. The diagnosis of cesarean scar pregnancy is primarily done via transvaginal or transabdominal ultrasound, and medical or surgical management can be used depending on the gestational age, hemodynamic status, and patient preferences. The surgical approach, which involves hysteroscopy, laparoscopy, or laparotomy, is usually preferred, since it is associated with fast recovery and lower recurrence rates. However, it is crucial to consider the patient’s plans for future fertility when selecting the management approach. In conclusion, cesarean scar pregnancy is a rare but potentially life-threatening condition that requires prompt and appropriate management. Early diagnosis and treatment can prevent significant morbidity and mortality, and surgical management is usually preferred due to its higher success rates and lower recurrence rates.

## 1. Introduction

Scar ectopic pregnancy is a rare type of ectopic pregnancy that is different from the more common tubal ectopic pregnancy, which occurs when the fertilized egg implants in the fallopian tube [1]. Scar ectopic pregnancy occurs when the fertilized egg implants in the scar tissue of a previous caesarean section or other uterine surgery, such as myomectomy or cornual resection [2]. The scar tissue can provide a less stable environment for the growing embryo, which can lead to a higher risk of maternal morbidity and mortality, as well as fetal loss [3].

Scar ectopic pregnancy has an estimated incidence of 1 in 1800 to 1 in 2214 pregnancies [4]. This is lower than the incidence of tubal ectopic pregnancy, which varies depending on patient populations and time. Some are estimated to be 1 in 100 pregnancies [5]; others are estimated at 20.70 per 1000 [6]. However, scar ectopic pregnancy is still considered a significant medical condition due to its potential for serious complications [7]. The risk factors for scar ectopic pregnancy include a history of previous cesarean sections or other uterine surgeries, such as myomectomy or cornual resection [8]. Women who have had multiple previous surgeries are at a higher risk of scar ectopic pregnancy compared to those who have had only one surgery [9]. Additionally, certain conditions that affect the shape or structure of the uterus, such as bicornuate uterus or uterine septum, can increase the risk of scar ectopic pregnancy [10].

Other risk factors for scar ectopic pregnancy include the use of assisted reproductive technology (ART) and older maternal age [11]. Women who conceive using ART may be at a higher risk of scar ectopic pregnancy due to the increased likelihood of uterine scarring from procedures such as in vitro fertilization (IVF) [12]. Older maternal age may also increase the risk of scar ectopic pregnancy due to the higher prevalence of uterine fibroids and other conditions that can cause uterine scarring [13]. It is important to note that, while these factors increase the risk of scar ectopic pregnancy, the condition can still occur in women without any known risk factors [14]. Therefore, a high index of suspicion is necessary in any woman with a previous uterine surgery who presents with symptoms of ectopic pregnancy [15].

Diagnosing scar ectopic pregnancy can be challenging, as the symptoms and signs are often similar to those of a normal pregnancy [16]. However, women who have had previous uterine surgeries, especially cesarean sections, should be monitored closely during early pregnancy for signs of scar ectopic pregnancy [17]. A high index of suspicion is necessary when a woman presents with abdominal pain, vaginal bleeding, or a positive pregnancy test after a previous uterine surgery [18]. Women who have a history of previous cesarean sections or other uterine surgeries should be evaluated for scar defects during prenatal care, and any suspicious symptoms should be promptly evaluated [19].

A transvaginal ultrasound is the preferred imaging modality for the diagnosis of scar ectopic pregnancy [20]. The ultrasound can show the location and size of the gestational sac, the presence of fetal cardiac activity, and the thickness of the myometrium around the scar [21]. The gestational sac may be seen within the cesarean section scar, and this is usually accompanied by a thinning of the myometrium in the scar area [22]. The presence of fetal cardiac activity within the gestational sac confirms the diagnosis of a viable pregnancy. In some cases, an MRI or hysteroscopy may be used for diagnosis. An MRI can provide more detailed information about the location and extent of the scar defect, while a hysteroscopy can be used to directly visualize the gestational sac within the scar [23]. It is important to diagnose scar ectopic pregnancy early to avoid potentially life-threatening complications [24]. Women with a suspected or confirmed diagnosis of scar ectopic pregnancy should be managed by a multidisciplinary team, including obstetricians, radiologists, and surgeons [25].

The management of scar ectopic pregnancy is complex and challenging, as there is no established standard of care for this condition [26]. Treatment options include expectant management, medical management with methotrexate, and surgical management with laparoscopy or laparotomy [27]. The choice of treatment depends on several factors, including the gestational age of the pregnancy, the size and location of the gestational sac, the presence of fetal cardiac activity, and the desire for future fertility [28].

Expectant management, also known as watchful waiting, may be appropriate for some cases of scar ectopic pregnancy, especially if the gestational sac is small and there is no evidence of fetal cardiac activity [29]. In this approach, the patient is monitored closely with regular ultrasound examinations to ensure that the pregnancy does not progress further and that there are no signs of rupture or hemorrhage. If there is no resolution of the pregnancy after a certain period of time, medical or surgical intervention may be necessary [30].

Medical management with methotrexate is another treatment option for scar ectopic pregnancy, especially for cases where the gestational sac is smaller, and the patient is hemodynamically stable [31]. Methotrexate is a chemotherapy drug that stops the growth of rapidly dividing cells, including the cells in the developing embryo. The drug is administered either intramuscularly or systemically, depending on the patient’s condition and the size of the gestational sac [32]. The success rate of medical management varies depending on the size and location of the gestational sac, and the presence of fetal cardiac activity [33].

Surgical management with laparoscopy or laparotomy is the most common treatment for scar ectopic pregnancy, especially for cases where the gestational sac is larger, there is evidence of fetal cardiac activity, or the patient is hemodynamically unstable [34]. A surgical approach involves removing the gestational sac from the scar tissue, while preserving as much of the healthy uterine tissue as possible [35]. The choice of surgical approach depends on several factors, including the size and location of the gestational sac, the experience of the surgeon, and the availability of resources [36].

## 2. Case Description

A 44-year-old female, G7 P6, presented to the emergency department with sudden onset of mild to moderate vaginal bleeding that began 3 h prior. The patient had a history of four cesarean sections, with the first one being due to breech presentation and the second one because of fetal distress. The third and fourth ones were elective cesarean sections. The gestational age of the patient was 7 weeks. The patient complained of on and off left lower quadrant pain that began three days prior.

**Figure 1:**
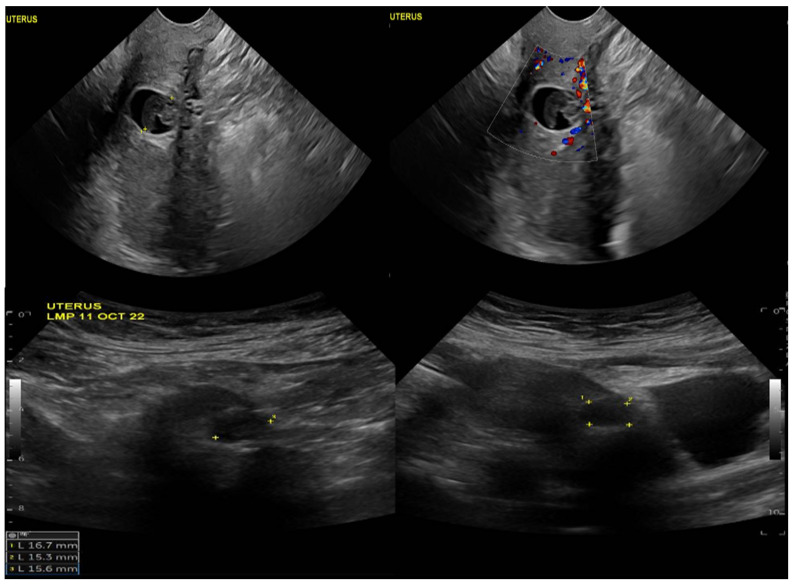
Ultrasound image of the uterus, demonstrating a well-defined ectopic gestational sac and showing a fetal pool with no definite pulsations in the anterior wall of the lower uterine segment at the assumed region of the previous scar.

Upon physical examination, the patient was vitally stable with no active bleeding. A speculum examination showed a healthy-looking vulva and vagina, no bleeding, and a closed cervical os. The patient’s B-HCG level was 6878 mIU/mL, which increased to 9355 mIU/mL when repeated. A transvaginal ultrasound showed an average-sized uterus measuring 8 × 4 cm, a homogenous myometrium, and an endometrium measuring 4 mm with no definite endometrial gestational sac (Figure 1). The ultrasound also revealed a well-defined ectopic gestational sac showing a fetal pool with no definite pulsations in the anterior wall of the lower uterine segment at the assumed region of the previous scar.

The patient was admitted for further management. Routine blood and urine investigations were normal. The impression was a lower uterine segment scar ectopic pregnancy showing a fetal pole corresponding to 7 weeks and 4 days of gestation with no detectable cardiac pulsation. The patient underwent an uncomplicated ultrasound-guided suction and curettage with an estimated blood loss of 50 mL. Tissue was sent for a histopathology examination, which showed decidua and chorionic villi consistent with a product of conception. Postoperatively, 24 h later, the patient’s B-HCG level dropped to 2085 mIU/mL, and she was discharged 1 day post-operation. The patient was scheduled for an in-clinic follow-up. On day 7 post-operation, the B-HCG level dropped to 37 mIU/mL.

## 3. Literature Review

### Materials and Methods

A comprehensive search was conducted on the PubMed database using the search terms “scar ectopic pregnancy [all fields]” and “cesarean scar pregnancy [all fields]” to identify relevant literature published in English from 2000 to 2022, including case reports and review articles. Additional references were identified through a manual search of the reference lists of relevant articles. All case reports of scar ectopic pregnancy were included in the review, and data on clinical presentation, diagnostic modalities, management strategies, and outcomes were collected and analyzed. Any reports of other types of ectopic pregnancy were excluded from this study.

## 4. Result

Utilizing the search strategy outlined above, a total of 113 pertinent English language documents were initially identified from various databases. Following a thorough review of the complete texts, a total of 34 articles with 24 unique cases were identified (as summarized in Table 1). In conjunction with this current case report, the cumulative dataset comprised a total of seven cases.

Based on the extracted data of the included studies [1,25,37,38,39,40,41], the diagnosis of scar ectopic pregnancy was confirmed using various diagnostic methods, such as a transvaginal ultrasound, MRI, and transvaginal ultrasound-guided puncture. The patients presented with symptoms such as abdominal pain, vaginal bleeding, and spotting.

The intervention methods varied and included an intragestational sac injection of methotrexate, laparoscopic resection, laparotomy, and dilatation and curettage. Following the intervention, all of the patients showed an improvement in their condition, and there were no clinical complications reported. The outcomes varied, but all showed successful management of the ectopic pregnancy, with a decline in β-human chorionic gonadotropin levels or an absence of the gestational sac observed during follow-up.

The age of the patients ranged from 25 to 44 years, with the majority being in their late twenties. The sample size is small, with only six case reports included. Nonetheless, this review provides useful insights into the diagnosis, management, and outcomes of scar ectopic pregnancy.

## 5. Discussion

Ectopic pregnancy accounts for 4% of pregnancy-related deaths and is the primary cause of morbidity and mortality in fertile women [42]. A cesarean scar ectopic pregnancy is one of the kinds of ectopic pregnancy where the embryo implants in the uterine scar, and it is considered the rarest among all ectopic pregnancies [43].

The etiology of cesarean scar ectopic pregnancy is explained by a wide variety of theories [2]. According to one theory, a blastocyst may implant in a uterine scar that has a minuscule dehiscent channel [44]. Any uterine operation, such as a myomectomy or even the manual removal of the placenta, could leave this scar [45]. As per another theory, even in the absence of prior uterine surgery, intramural implantation is still possible after in vitro fertilization and embryo transfer [46]. A fibrinoid degeneration layer or decidua basalis deficiency can be found in uterine scars. This ectopic pregnancy implants into myometrium and fibroid scar tissue rather than being surrounded by a decidualized endometrium. This pregnancy is one that is unusual from the beginning and needs particular care [47].

The majority of ectopic pregnancies caused by cesarean scars are asymptomatic [48]. A small number of people can manifest with mild abdominal pain or light vaginal bleeding. An ectopic pregnancy caused by a cesarean scar does not have any pathognomonic signs or symptoms [49]. Based on the criteria outlined in GTG 2112 (Green-top Guideline No. 21), a transabdominal ultrasonography may be used in addition to a transvaginal ultrasound as the primary modality for diagnosis [50]. The two main management modalities are medical and surgical. For pregnancies with cesarean scars, as well as other types of ectopic pregnancies, medical therapy with injection methotrexate has been utilized successfully [51].

Candidates for methotrexate are pregnancies with gestational ages of eight weeks that are symptom-free, hemodynamically stable, and unruptured [52]. Methotrexate prevents cell division. It can be administered intramuscularly—systemically, locally, or in a combination of both. Methotrexate, potassium chloride (KCL), hyperosmolar glucose, or crystalline trichosanthin have all been injected once under ultrasound guidance [53]. Systemic regimens for cesarean scar pregnancy have produced reassuring outcomes, both with and without intrasac medication injections. There have been single-dose and multidose procedures employed [54]. Methotrexate is typically administered as a single dosage of 50 mg/m^2^, but the multidose protocol is to administer four doses of the drug—1 mg/kg each given on days 1, 3, 5, and 7—along with 0.1 mg/kg of folinic acid on alternate days [55].

Systemic methotrexate seems to work best for patients with ectopic pregnancies and hCG levels under 5000 mIU/mL. If significant bleeding develops, close monitoring is necessary and may need to be coupled with surgical techniques, either electively or urgently [56].

Hysteroscopic suction evacuation and curettage, a laparoscopic or open excision of the scar while pregnant, and hemostatic procedures like the use of a double balloon catheter for tamponade and uterine artery embolization are all surgical management possibilities [57]. Numerous authors support surgery even when there is no bleeding at that moment. It entails a laparotomy, scar removal, and resuturing to lessen recurrence and shorten the follow-up period [58,59]. As a first line of treatment, uterine curettage should be avoided since it may cause bleeding, uterine rupture, or failure to reach the fetus [60]. However, it can be used in conjunction with hysteroscopy when done under direct vision, especially following a successful course of medical treatment [60]. The laparoscopic method and transvaginal approach can be combined. Hysteroscopy and laparoscopy were combined in a case series by Ash et al. Prior to hysteroscopy, a laparoscopy was conducted to divide the bladder peritoneum from the lower uterine segment to remove the bladder from the site of surgical management and reduce the risk of harm if the anterior myometrial thickness measured on ultrasound was less than 3 mm. In this case series, 44 individuals had their sperm and eggs removed successfully [61].

Other surgical alternatives include uterine artery embolization, which lowers the risk of bleeding by injecting an embolus into the uterine arteries before hysteroscopy [62]. However, patients’ plans for future fertility should be taken into account, because this surgery may unintentionally result in ovarian embolization [63]. Additionally, a double balloon catheter might be employed to stop the bleeding. Surgical treatment is superior to medical treatment and is associated with fast recovery. More importantly, sharp curettage should be avoided [64,65]

## 6. Conclusions

This literature review and case report provide valuable insights into the diagnosis and management of caesarean scar ectopic pregnancy, a rare but potentially life-threatening complication of pregnancy. The review highlights the challenges in diagnosing and treating this condition and emphasizes the importance of early detection and prompt intervention to prevent serious morbidity and mortality. The case report illustrates the successful management of a caesarean scar ectopic pregnancy, using a combination of medical and surgical modalities and considering the patient’s future fertility goals. Overall, these findings underscore the importance of raising awareness about this rare but critical condition and developing optimal management strategies to improve outcomes for affected patients.

## Figures and Tables

**Table 1:** The extraction table of the included studies.

References	Age	Diagnosis Method	Symptoms	Intervention	Outcome
(Al Gadeeb et al., 2019) [37]	44	Positive urine testing, transvaginal ultrasound examination, Magnetic resonance imaging (MRI).	Abdominal pain.	Intragestational sac injection of methotrexate.	The serum β-human chorionic gonadotropin level was undetectable on the 35th day after the methotrexate injection.
(Thakur & Shrimali, 2023) [1]	27	Pelviabdominal ultrasonography (U.S.G), which suggested a very early cesarean scar ectopic pregnancy.	1.5 months of amenorrhea and complaints of vaginal bleeding that lasted two days.	Under spinal anesthesia, dilatation and curettage were carried out. A surgically removed product of conception (POC).	After surgery, the patient’s condition improved, and she was discharged.
(Majangara et al., 2019) [25]	36	Transvaginal ultrasound imaging.	Abdominal pain.	Ectopic gestation was excised, and the uterus was repaired via laparotomy.	Improvement of the case.
(Yadav et al., 2022) [38]	27	b-hCG levels were 38,075.6 IU/L. Transvaginal ultrasonography was performed, which revealed a single gestational sac with a mean sac diameter of 2.7 mm, corresponding to the gestational age of four weeks and five days which was seen in lower uterine segment eccentric to the location of the previous scar site.	1½ months amenorrhea with chief complaints of pain in abdomen and per vaginum spotting for three days.	The patient underwent laparoscopic resection.	A post procedure transvaginal ultrasound showed an absence of the gestational sac, and the patient was discharged without any complaints on postoperative day 2. At follow-up, the patient’s β-hCG level was 4 mIU/mL after 1½ months of her treatment.
(Kharode et al., 2022) [39]	39	Transvaginal sonography indicated an empty uterine cavity with well-defined endometrium.	Amenorrhea for 1.5 months; bleeding p/v (spotting) for 8 days.	Laparotomy.	Improvement of patient bleeding and abdominal pain.
(Deepika et al., 2017) [40]	25	Routine blood and urine investigations were normal. On admission, B-HCG level was 7118 IU/L, and after 48 h, the B-HCG value was 8108 IU/L, which showed less than doubling. Trans vaginal ultrasound revealed an empty uterine cavity; MRI of the pelvis showed a poorly defined heterogenous signal intensity space-occupying lesion of 30 × 23 mm, seen in myometrium.	Two-month amenorrhea with bleeding per vaginum on and off for 10–12 days.	Patient was planned for laparotomy.	Patient was followed up with serum Beta human Chorionic Gonadotropin (ß-hCG) level until B-HCG reached a non-pregnant level.
(Leite et al., 2016) [41]	30	β-hCG of 18,716, mIU/mL, Transvaginal ultrasound performed on this day showed a 6 mm crown to rump length (CRL), corresponding to a gestational age of 6 weeks and 4 days, a gestational sac of 16 × 14 × 9.6 mm located at the site of the previous cesarean section scar with a live embryo (126 bpm fetal heart rate), an empty uterine cavity, and attachments without changes.	6 weeks late on her period, although presenting a small amount of vaginal bleeding for 1 day.	Transvaginal ultrasound-guided puncture and injection of methotrexate inside the gestational sac at 68 mg (1 mg/kg).	The patient remained in hospital until the last dose of folinic acid without clinical complications after the procedure, showing only slight abdominal colic treated with analgesia.

## Data Availability

Data are available upon request.
